# Biologics in T2 Severe Asthma: Unveiling Different Effectiveness by Real-World Indirect Comparison

**DOI:** 10.3390/jcm13164750

**Published:** 2024-08-13

**Authors:** Elisa Riccardi, Giuseppe Guida, Sonia Garino, Francesca Bertolini, Vitina Carriero, Mattia Brusamento, Stefano Pizzimenti, Fabiana Giannoccaro, Erica Falzone, Elisa Arrigo, Stefano Levra, Fabio Luigi Massimo Ricciardolo

**Affiliations:** 1 Regional Hospital Parini, Pulmonology Unit, Aosta, 11100 Aosta, Italy; eli16riccardi@gmail.com; 2 Department of Clinical and Biological Sciences, University of Turin, Orbassano, 10043 Turin, Italy; sonia.garino@unito.it (S.G.); francesca.bertolini@unito.it (F.B.); vitina.carriero@unito.it (V.C.); erica.falzone@unito.it (E.F.); elisa.arrigo@unito.it (E.A.); stefano.levra@unito.it (S.L.); fabioluigimassimo.ricciardolo@unito.it (F.L.M.R.); 3 Severe Asthma, Rare Lung Disease and Respiratory Pathophysiology, San Luigi Gonzaga University Hospital, Orbassano, 10043 Turin, Italy; pizzimentistefano@gmail.com (S.P.); fabiana.giannoccaro@gmail.com (F.G.); 4 Re Learn S.R.L., 10122 Turin, Italy; bmattia92@gmail.com; 5 Institute of Translational Pharmacology, National Research Council (IFT-CNR), Section of Palermo, 90146 Palermo, Italy

**Keywords:** severe asthma, indirect comparison, biologics, omalizumab, mepolizumab, dupilumab, benralizumab, precision medicine, treatable traits

## Abstract

**Background**: Indirect comparison among biologics in severe asthma (SA) is a challenging but desirable goal for clinicians in real life. The aim of the study is to define characteristics of a biologic-treated T2-driven-SA population and to evaluate the effectiveness of biologic treatments in a real-world setting by variation in intra/inter-biologic parameters in an up to 4-year follow-up. **Methods**: Demographic, clinical, functional, and biological characteristics were evaluated retrospectively in 104 patients recruited until July 2022 at baseline (T0) and over a maximum of 4 years (T4) of biologic therapy (omalizumab/OmaG = 41, from T0 to T4, mepolizumab/MepoG = 26, from T0 to T4, benralizumab/BenraG = 18, from T0 to T2, and dupilumab/DupiG = 19, from T0 to T1). Variations of parameters using means of paired Delta were assessed. **Results**: At baseline, patients had high prevalence of T2-driven comorbidities, low asthma control test (ACT mean 17.65 ± 4.41), impaired pulmonary function (FEV_1_ 65 ± 18 %pred), frequent exacerbations/year (AEs 3.5 ± 3), and OCS dependence (60%). DupiG had lower T2 biomarkers/comorbidities and AEs, and worse FEV_1_ (57 ± 19 %pred) compared to other biologics (*p* < 0.05). All biologics improved ACT, FEV_1_%, FVC%, AEs rate, and OCS use. FEV_1_% improved in MepoG and BenraG over the minimal clinically important difference and was sustained over 4 years in OmaG and MepoG. A significant RV reduction in OmaG (T4) and DupiG (T1), and BenraG normalization (T2) of airflow limitation were found. We observed through inter-biologic parameters pair delta variation comparison a significant nocturnal awakenings reduction in BenraG vs. OmaG/MepoG, and neutrophils reduction in BenraG/DupiG vs. OmaG. **Conclusions**: Indirect comparison among biologics unveils clinical and functional improvements that may mark a different effectiveness. These results may highlight the preference of a single biologic compared to another with regard to specific treatable traits.

## 1. Introduction

Severe asthma (SA) is a complex and heterogeneous disease presenting several clinical phenotypes driven by multiple molecular endotypes and affecting 5–10% of asthmatic patients [1]. At present, asthma phenotypes can be divided into two main groups based on the underlying inflammatory process: Type-2 (T2) High, representing approximately 70% of SA cases and associated with an eosinophilic inflammatory profile in induced sputum, and T2-Low [2]. The majority of all currently approved biologics for uncontrolled, moderate-to-severe asthma, target components of the T2 inflammatory pathway. Omalizumab suppresses the activity of IgE, mepolizumab binds IL-5, benralizumab blocks IL5Rα, whereas dupilumab inhibits the activity of IL-4 and IL-13. All biologics reached, in both a randomized controlled trial (RCT) and real-life studies (RLS), the expected outcomes in reducing airway eosinophilic inflammation, asthma exacerbation (AEs) rates, and improving lung function and symptoms scores [3]. Only recently, tezepelumab, a human monoclonal antibody that binds specifically to thymic stromal lymphopoietin (TSLP) and targets multiple disease pathways, including T2-low severe asthma, was adopted in clinical practice [4].

Although designed on different biologic targets, the indications for the clinical use of biologics to severe, T2, asthmatic patients frequently overlap, turning the choice for the best biologic treatment into a challenge [5]. At present no direct “head-to-head” trials of comparison between biologics in SA are available, while indirect methods, such as indirect treatment comparison (ITC), were explored. They compare the efficacy of each treatment based on selected endpoints in cohorts of patients with same defined selected clinical and inflammatory phenotypes, using different statistical methods. None of the studies using ITC succeeded in matching patient characteristics and many can be criticised because of arbitrary inclusion and exclusion criteria [6]. Moreover, ITC relied strictly on controlled data only from RCTs; accordingly, they are not generalizable and may underestimate the true treatment efficacies [7]. Recently, Taha Al-Shaikhly and colleagues demonstrated the relatively superior efficacy of Dupilumab in reducing AEs compared with anti-IL-5 and anti-IgE biologics in real life. However, head-to-head controlled RLS are still needed [8].

The great heterogeneity of T2 SA population supports the existence of distinct subtypes of T2 SA which could preferentially respond to a single biologic [9,10]_._ Pragmatic algorithms to guide the choice of biologic based on sub-endotypes of T2 asthma were suggested, remaining largely speculative from an evidence-based perspective [11]. With the upcoming of a great deal of data coming from RLS, the concept of clinical treatable traits within the T2, SA patients emerged, allowing a precision medicine approach [12]. A treatable trait is defined as a phenotypic or endotypic characteristic that can be successfully targeted with treatment. Each trait, such as comorbidities, lung function, or asthma symptoms could be a preferential target for one specific biologic. Thereafter, biomarkers in SA were explored with the aim of identifying the treatable trait and prediction of response to treatments [13]. Concomitantly, molecular phenotyping validated the recognition of biological endotypes that represent treatable mechanisms which need to be linked to biomarkers according to precision medicine approaches [14].

We aimed to compare retrospectively clinical, functional, and biological characteristics in a cohort of SA patients before and during treatment with four different biologic agents (omalizumab, mepolizumab, benralizumab, or dupilumab) in order to bring out those traits marking a different effectiveness. The evaluation of parameter variations over time for each biologic lets us define the “intra-biological” and the “inter-biological” changes in real life as a measure of indirect comparison.

## 2. Materials and Methods

### 2.1. Patients and Study Design

This monocentric, retrospective, observational, and real-life study was conducted at San Luigi Gonzaga University Hospital with the approval of the local ethical committee (Protocol number 4478/2017, approved on 20 March 2017) in accordance with the Declaration of Helsinki. The study involved 88 SA patients who gave written informed consent and who accessed our Severe Asthma and Rare Lung Disease Unit from January 2007 to July 2022. SA was defined according to ATS/ERS Guidelines [1]. All patients presented with T2 inflammation and were prescribed a biologic agent according to regional criteria for prescription (Table S1). T2 inflammation was defined if at least one of the following elements were present: peripheral blood eosinophils (PBE) ≥ 300/mcl, F_E_NO ≥ 30 ppb, total IgE ≥ 100 UI/mL, or documented atopy through *prick test* or specific IgE measurement [15]. Patients were divided into four groups based on the biologic prescribed: omalizumab group (OmaG), mepolizumab group (MepoG), benralizumab group (BenraG), and dupilumab group (DupiG). Four patients included in the study were treated off-label with omalizumab, due to lack of available alternative biologics in the market at the time of prescription.

### 2.2. Baseline Descriptive Clinical, Functional, and Biological Characteristics

For each patient, we reported the following data: age, sex, age of asthma onset (<18 y/o: early-onset/>18 y/o: late-onset), BMI, history of smoke, comorbidities, atopy for seasonal or perennial allergens (animal dander or house dust mites), atopy for molds demonstrated by diagnostic tests (prick or specific IgE), HRCT characteristics (bronchiectasis, mucus plug, emphysema, and thickening of the bronchial walls), ER visits for asthma exacerbations, intubation due to asthma attacks, and number of exacerbations that required OCS burst in the previous year. We also reported maintenance treatment defining ICS dose, OCS dose, LABA, and LAMA. ICS was expressed as beclomethasone equivalent HFA (BDP HFA dose, mcg). OCS dependence patients were defined as patients who have at least one between the following characteristics: need of chronic treatment with OCS for more than 6 months in the previous year (chronic OCS) or number of asthma exacerbations that required at least 3 days of treatment with OCS ≥ 3/year in the previous year (OCS bursts ≥ 3/year). To assess asthma symptoms, an asthma control test (ACT) [16] was proposed to each patient at every follow-up visit. Activity limitations and nocturnal symptoms were evaluated through the first two questions of the ACT. Asthma was defined as “non controlled” if ACT score was ≤19 and as “controlled” if ACT was ≥20 [17]. Pulmonary function was assessed performing spirometry and/or plethysmography (Vmax Encore 62, Carefusion, Würzburg, Germany) with or without a post-bronchodilator test. The following spirometric data were collected: absolute FEV_1_, FEV_1_ %pred., absolute FVC, FVC %pred., absolute IT, IT %pred., absolute RV, RV %pred., absolute FVC post BD, absolute Delta FVC post BD, Delta FVC %post BD, absolute FEV_1_ post BD, absolute Delta FEV_1_ post BD, Delta FEV_1_ %post BD, DLCO %pred., and DLCO/Va %pred. We also evaluated the percentage of patients that showed reversibility of FEV_1_ (reversible) and the percentage of patients with fixed obstruction of the Tiffeneau index, after a bronchodilator test in accordance with ATS/ERS Guidelines [18]. Biological collected data included total IgE (UI/mL), total (cells/mcl), and percentage count of leukocytes, neutrophils, peripheral blood eosinophils PBE, fibrinogen levels (mg/dl) [19], and F_E_NO values (ppb). F_E_NO was measured with the single breath technique using F_E_NO + (Medisoft, Sorinnes, Belgium). The presence of one or more biomarkers defining T2 inflammation, as defined in the methods section, was analyzed for each group of biologic-treated patients.

### 2.3. Collection of Variables for “Intra and Inter Biologics” Comparison over Time

We evaluated the variation in clinical, functional, and biological continuous variables in patients who were prescribed a biologic over 4 years (T1 = first year, T2 = second year, T3 = third year, and T4 = fourth year) in OmaG and MepoG, 2 years (T1 and T2) in BenraG, and 1 year (T1) in DupiG. OCS chronic treatment discontinuation or reduction over years was assessed.

Data from patients that switched from a biologic to another or more (N = 16) were collected before each start, so that a patient could be considered more than once in the analysis of comparison within and among biologics over years (N = 104). A wash-out period of 3 months was considered.

### 2.4. Statistical Analysis

Descriptive analysis and baseline comparisons were analysed using Graph Pad Prism software (version 9.0; GraphPad Software Inc., San Diego, CA, USA) and SPSS Statistic Version 28 (IBM Corp, Armonk, NY, USA). Descriptive analysis results are expressed as means ± SDs for continuous variables and as number/percentage for categorical variables. Python Version 3.8 was used for the A T paired sample test to evaluate the variation in Delta parameters for each year of treatment. The normality of the distributions was evaluated with D’Agostino and Pearson Test. The ROUT method detected outliers to be excluded. The Anova test (with Tukey post hoc test) or Kruskal–Wallis H-test (with Dunn post hoc test) were used to compare continuous variables, while the F Fisher test is used to compare categorical variables. Welch T test was performed to compare parameters at baseline between biologics, over years for each biologic and over years between biologics. *p* values of less than 0.05 were considered statistically significant.

## 3. Results

### 3.1. General Characteristics at Baseline of Severe Asthma Biologic-Treated Patients

The summary of general baseline characteristics is reported in Table 1 and Table 2. At baseline the analysis of demographic characteristics did not show any significant difference between the groups.

OmaG were all atopic with prevalent polysensitization to both seasonal and perennial allergens while it was about 50% for the other groups with a very low number (11.1%) of polysensitized in DupiG (*p* < 0.00001).

Concerning comorbidities, OmaG had less bronchiectasis on chest HRCT compared to BenraG (*p* < 0.05), less prevalence of obesity and GERD (*p* < 0.05) compared to MepoG, and presented a more controlled asthma of MepoG and DupiG (*p* < 0.01). DupiG had less rhinitis compared to other groups (*p* < 0.001) and a lesser mean number of AEs (*p* < 0.05 vs. MepoG). BenraG took higher doses of ICS compared to OmaG and MepoG (*p* < 0.05).

At baseline, DupiG had worse pulmonary function compared to OmaG and BenraG. In particular, FVC abs. and FEV_1_ abs/ %pred. both pre and post BD were significantly inferior in DupiG (*p* < 0.05) compared to BenraG, while FEV_1_ abs. pre and post BD were significantly inferior compared to OmaG (*p* < 0.05).

Lower basal F_E_NO values compared to MepoG (*p* < 0.05) and lower count of PBE as compared to others (*p* < 0.05) were reported in DupiG.

### 3.2. T2 Phenotyping Patients

The majority of patients (86.3%) had at least two positive T2 biomarkers at baseline. DupiG patients reported a significantly lower rate of 3–4 T2 positive biomarkers compared to BenraG and MepoG *(*Table 3).

### 3.3. Analysis of “Intra-Biologic” Parameters over Years

The number of SA patients for each bio group decreased over time, as shown in Table S2, for lost in follow-up, outbreak of pandemic SARS-CoV-2, or different starting point of biologics. The number and sequence of switches from a biologic to another is represented in Figure S1.

As shown in Table 4, OmaG showed a significant reduction in ICS mean dose (*p* < 0.05) at T4, an improvement in ACT, a significant reduction in AEs (from 3.15 to 1.0; *p* < 0.001 at T1), and an increase in FEV_1_ and FVC % pred. already evident from T1 (from 65.08 to 72.87 and from 89.02 to 97.02, respectively *p* < 0.05). A reduction in RV %pred. emerged at T4 (from 159.11 to 113.0, *p* < 0.05). Anti-IgE did not affect F_E_NO values nor PBE count, while total IgE levels increased.

MepoG showed from T1 a significant progressive improvement in ACT (*p* < 0.000.1 at T3), activity limitations (*p* < 0.0001 at T3), and nocturnal symptoms (*p* < 0.05). MepoG dramatically reduced the number of AEs at T1 (from 4.27 to 1.08, *p* < 0.0001) with greater effects to T4 (0.44) and improved pulmonary function starting from T2 with the highest value at T3 (∆FEV_1_ %pred. 24%; *p* < 0.01, absolute delta +610 mL, and FVC %pred. *p* < 0.00001). At T4 post-FVC BD reversibility decreased (*p* < 0.001). MepoG significantly reduced PBE count at T1 (*p* < 0.00001) with further reduction over years, while it did not affect F_E_NO values nor total IgE.

BenraG increased ACT at T1 (*p* < 0.05) and reduced AEs (4.0 vs. 0.89, *p* < 0.001). An improvement of FEV_1_ (+850 mL/+25.4%) and IT (from 60.2 to 72.0) was clearly evident at T2 with normalization of IT abs. (*p* < 0.05). Anti IL5-Rα did not influence F_E_NO or total IgE values, while it reduced PBE count starting from T1 with a total suppression at T2 (*p* < 0.00001). We also observed a significant mean reduction in the neutrophils absolute count at T2 (from 4570 to 3115, delta −955/mcl; *p* < 0.001) and a trend toward a concomitant reduction in fibrinogen values (from 327.3 to 294.0).

DupiG improved ACT score, activity limitations and nocturnal symptoms (*p* < 0.001) at T1. It was able to decrease the number of AEs (2.58 vs. 0.42, *p* < 0.0001) and RV abs (delta −660 mL; *p* < 0.05). An improvement in delta changes in FEV_1_ was observed, although not statistical significance (+260 mL, +7.26%). It did not affect F_E_NO or IgE values while showing an increase in PBE count which was not statistically significant.

### 3.4. Analysis of “Inter-Biologic” Parameters over Years

Table 5 shows the “inter-biologic” comparison of means of paired Delta change for each variable over years.

**Figure 1:**
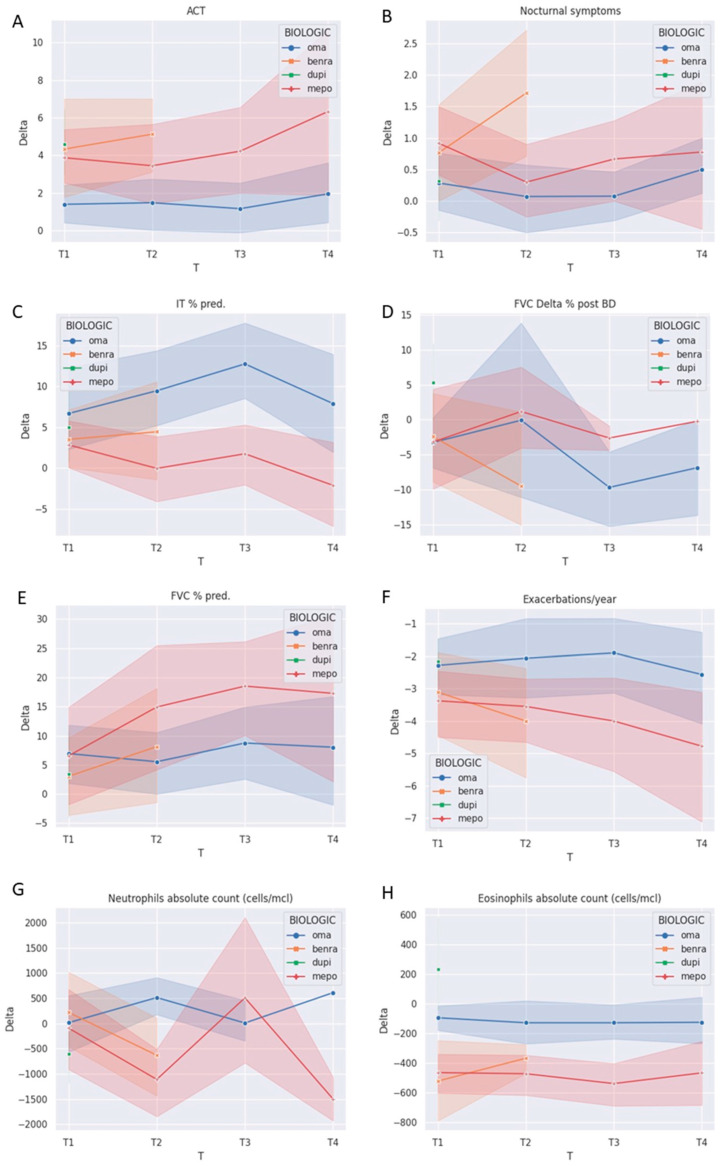
Solid lines represent the trend of mean paired Delta values calculated from each time point towards T0. Shaded areas cover 95% IC. (**A**) ACT: asthma control test, (**B**) nocturnal symptoms, (**C**) TI % pred.: Tiffeneau index, (**D**) FVC Delta % post BD, (**E**) FVC% pred., (**F**) exacerbations/year, (**G**) neutrophils, and (**H**) eosinophils absolute count.

BenraG and DupiG improved ACT at T1 more than OmaG (*p* < 0.05, *p* < 0.001, respectively), while MepoG improved ACT at T3 more than OmaG (Figure 1A). BenraG reduced nocturnal asthma symptoms more than both OmaG and MepoG at T2 (*p* < 0.05, Figure 1B). The AEs reduction was similar among biologics but more pronounced at T3 in favor of MepoG vs. OmaG (*p* < 0.05, Figure 1F) of MepoG than DupiG at T1. OmaG improved more IT% pred. from T2 to T4 (*p* < 0.01 at T3) compared to MepoG with a trend of amelioration with regard to FVC %pred. in favor of MepoG to OmaG at T3 (Figure 1C, E). Only DupiG showed an improved FVC Delta% post BD at T1 compared to OmaG (Figure 1D). We observed a significant downward trend in the neutrophil count both for BenraG and DupiG compared to OmaG at T1 (Figure 1G, *p* < 0.05). Finally, PBE was significantly reduced starting from T1 for MepoG and BenraG while augmented in DupiG (Figure 1H).

### 3.5. OCS Chronic Treatment

**Figure 2:**
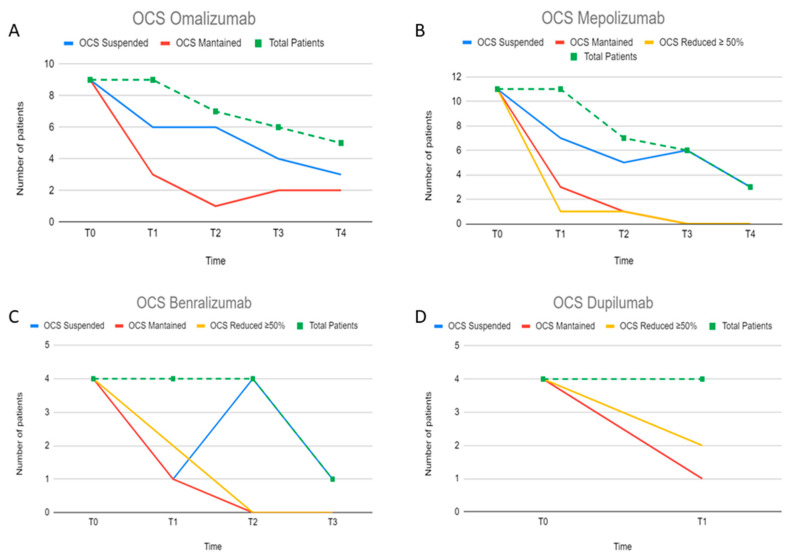
(**A**–**D**) Graphics showing for each biologic agent the number of patients who suspended/maintained OCS chronic treatment over years (T0–T4). (**A**) Omalizumab, (**B**) mepolizumab, (**C**) benralizumab, and (**D**) dupilumab.

At baseline, there were no differences in the prevalence of patients with chronic OCS treatment (Table 1). Overall, at T1, discontinuation of OCS was reached of 16/31 (51.6%), with the best yield for OmaG (66%) and MepoG (63.6%). At T4, 60% (N = 5) of patients in the OmaG withdrew OCS, while all patients interrupted OCS maintenance treatment at T3 in MepoG and at T2 in BenraG. The proportion of patients interrupting chronic OCS in DupiG was 28.5% at T1, while 28.5% reduced chronic OCS therapy by 50% (Figure 2A–D and Table S3).

## 4. Discussion

This retrospective study compared clinical, functional, and biological characteristics in a cohort of SA patients before and during treatment with omalizumab, mepolizumab, benralizumab, and dupilumab. Baseline characteristics of SA biologic-treated patients revealed that DupiG likely included more patients with mixed phenotype (77% of patients with only 1 or 2 T2-positive biomarkers) compared to other groups, maybe due to less stringent prescription criteria with respect to T2 biomarkers. Actually, DupiG had less rhinitis and high GERD at baseline. The “intra-biologic” analysis confirmed, as in other RLS, the effect of all biologics on the expected outcomes; in all the four groups, AEs decreased significantly, ACT, FEV_1_%, and FVC% improved, while OCS were progressively withdrawn from T1 to T4.

In our study, omalizumab ameliorated ACT, although not reaching MID (improvement of ≥3) [20] activity limitations and decreased AEs already at T1. We observed a lung function improvement from T1 and RV reduction at T4, in line with observations from the INNOVATE study and RLS [21,22]. Other clinical observations regarding RV reduction are inconclusive [23]. Omalizumab seems to have a “deflating” action that occurs after a prolonged period of therapy (T4). In fact, IgE stimulates bronchial epithelial cells to synthesise growth factors involved in airway remodeling, such as TGF-β, smooth muscle cells proliferation, the release and production of pro-inflammatory agents, extracellular matrix proteins, and the synthesis of type I and III collagen [24,25]. We also observed progressive FVC improvement, but FVC Delta abs./% post bronchodilator reduced, which is explained by the limited further “reversibility” effect after normalization of lung function. Omalizumab did not affect F_E_NO values nor PBE count. Anti-IgE real-life experiences are now more than 5 years and, in some studies, associated with an observed F_E_NO reduction. However systematic review showed conflicting data on F_E_NO modulation by omalizumab, remaining unclear [26]. The increase in total IgE, occurred due to the formation of IgG-IgE immune complexes, which are erroneously considered in the count by the automatic counter.

Mepolizumab showed a significant effect on ACT already at T1, which was long lasting until T4. The effect on AEs reduction was dramatically positive from T1 with further improvement up to T4, in line with both RCT and RLS results [27,28,29,30]. We showed an increase already at T2 in FVC absolute value. The effect of mepolizumab on lung function is controversial and generally slight. According to MENSA and MUSCA trials, mepolizumab improved FEV_1_ when compared to placebo at 24 and 32 weeks, respectively [27,31], while in RLS, it showed an increase in FEV_1_ abs. of 230 mL at 12 months [32]. Although not statistically significant, the progressive decrease in RV suggests a slow improvement in dynamic hyperinflation. This anti-remodeling action is likely due to the reduction in TGF-β1 eosinophil BAL-derived synthesis mediated by mepolizumab [33]. ICS, F_E_NO, and total IgE values did not variate over years. In RLS, the effect of mepolizumab on F_E_NO was slow and mild with a mean reduction of 14.33 ppb [32] but, similarly to the current study, it was not evident in other studies [34].

In what regards benralizumab, ACT and AEs ratio improved already at T1. At T2, a significant increase in FEV_1_ and IT was evident. Despite RCT reporting an increase in FEV_1_ ranging from 80 mL at 3 months to 125 mL at 14th months [35,36], RLS extended this finding to +300 mL and +400 mL improvement at 48 and 96 weeks, respectively [37]. We confirmed this evidence reporting even a more pronounced effect at T2, this corresponding to a reversion from fixed obstruction to normal function (IT from 60 to 72). To our knowledge, our observation is one of the few regarding normalization of lung function with biologics [38]. These results prove the role of IL-5 in guiding the SA demodeling effect [39]. Total F_E_NO and IgE values did not change significantly during treatment, while PBE reduced to zero at T2, as expected. A significant mean reduction in neutrophil count and fibrinogen value was observed at T2, suggesting an anti-inflammatory long-term effect of Benralizumab. Recent studies demonstrated that IL-5R shares the β-chain with the GM-CSF receptor and was found on neutrophils infiltrating lungs and other anatomical sites of mice as well as on neutrophils in the BAL of children with refractory asthma [40,41]. We hypothesised that neutrophils reduction might be explained by benralizumab-induced direct killing of these cells through FcγRIIIa receptor-mediated binding to NK cells, and by GM-CSF receptor inhibition at a progenitor cell level.

The observation of dupilumab was limited at 1 year. ACT improvement was significant, as well as the AEs reduction, in line with previous RLS and RCT [29,42,43]. An improvement in lung function is present with a concomitant significant reduction in absolute RV. Based on our observation, dupilumab may have a predominant effect in demodeling, blocking IL-13 pathways. IL-13 causes contraction and proliferation of smooth muscle cells and is the main inducer of subepithelial fibrosis due to fibroblast proliferation and collagen production [44]. Surprisingly, at T1, no significant improvement in F_E_NO was observed. However, approximately 50% of DupiG are “switchers” with possibly reduced baseline F_E_NO values compared to naïve patients.

Here, we add during our Intra-biologic observation relevant and new effects on lung function. Some biologics give an improvement in FEV_1_% over the minimal clinically important difference [45] or are sustained for a very long time. Others have desufflating effects, as suggested from RV improvement. In our study, all patients received a high ICS dose at baseline (Table 1) that remained unchanged during the follow-up, therefore not significantly influencing the effects of biologics on lung function.

The “inter-biologic” analysis by comparing means of paired Delta change for each variable represents an indirect method of comparison. It is, to our knowledge, a never-explored method of comparison among biologics in SA. Benralizumab and dupilumab improved ACT at T1 and mepolizumab at T3, more than omalizumab, probably because OmaG showed higher ACT values at baseline. In addition, benralizumab reduced nocturnal asthma symptoms more than both omalizumab and mepolizumab at T2, this could be considered a specific “biologic-treated treatable trait”. The effect on AEs reduction is significant for all biologics with the only differences at T3 in favor of mepolizumab vs. omalizumab and at T1 of mepolizumab vs. dupilumab. This latter finding can be explained by the presence of numerous “switchers” in DupiG, presenting at baseline a lesser number of AEs. Dupilumab improved FVC Delta% post BD at T1 more than OmaG, sustaining the anti-remodelling action on small airways with partial recovery of FVC reversibility post BD [44]. On the contrary, benralizumab-induced increase in RV at T1 compared with OmaG, could be explained by a lesser effect on small airway disease in severe asthma in a real-life setting [46].

Discontinuation of OCS chronic treatment is an expected goal of biologic treatment in SA. Overall, it was reached in about 50% of patients. OmaG discontinued OCS up to T4 in 60% of patients. As far as RLS are concerned, approximately 50% of patients discontinued OCS chronic therapy at 1 or 4 years [22,47]. In what regards mepolizumab, at T1, 63.63% of patients discontinued OCS, up to 100% at T3. The OCS-sparing effect of mepolizumab is attested also in RLS with a 62% chronic OCS-treated patients reduction at 2 years [33]. In our study, the discontinuation rate at T3 is higher than data reported in literature. All BenraG OCS-treated patients discontinued therapy at T2, confirming the 82% complete OCS cessation at 36 months of therapy previously observed [30]. Here, only 28.5% DupiG suspended OCS at T1, definitively less than expected [43]; it is likely that OCS maintenance in this cluster is a clue of a more difficult-to-treat asthma in a potentially overlapped phenotype, often switching from an unsuccessful different biologic [8].

Our study presents some limitations. First, the present study is retrospective. Results from RLS of biologic-treated SA patients are strongly dependent on the population selected and on the physician attitudes in the choice of treatment. Patients’ and physicians’ preferences may regard less frequent dosing, SC administration, and faster onset, as well as cost/insurance coverage and convenience issues. In the present study, the group of physicians was the same over years and patients were always involved in the treatment choice [48]. The choice of a biologic was not generally guided according to predefined specific biomarker level (although the presence of high blood peripheral eosinophils may often lead to the use of IL-5 or IL-5R inhibitors, as well as high F_E_NO to anti IL4/IL-13 R). The reduction in the number of patients over years due to different starting time, loss in follow-up (pandemic SARS-CoV-2), or interruption, has limited statistical yield.

## 5. Conclusions

Our study underlined the differential beneficial effects of biologic treatments towards peculiar clinical, functional, and biological outcomes over years. The particularity of this work resides in the comparison between biologics using means of paired Delta, an indirect method of comparison able to unveil the superiority of a peculiar pathway targeting treatment in regard to a specific “trait”. This method could be useful to identify a specific “biologic-treated treatable trait” that can guide the choice among different biologics at baseline. The identification of different patient groups or traits with greater expected efficacy for a biologic remains as one of the greatest unmet needs in SA treatment.

## Figures and Tables

**Table 1:** Baseline demographic and clinical characteristics of patients treated with different biologics. Results are expressed as mean ± standard deviation or as number with relative percentage.

Demographic Characteristics					
	Overall	Omalizumab	Mepolizumab	Benralizumab	Dupilumab
**N Patients (%)**	88 (56.05%)	41 (46%)	23 (26.1%)	15 (17%)	9 (10%)
**Sex: female n (%)/male n (%)**	38(43.2%) /50 (56.8%)	16(39.0%) /25(61%)	10(43.5%) /13(56.5%)	9(60.0%) /6(40%)	3(33.3%) /6(66.7%)
**Age (Years)**	62.58 ± 11.92	64.24 ± 16.58	65.68 ± 14.82	60.87 ± 10.54	69.56 ± 16.0
**BMI (Kg/m^2^)**	27.07 ± 5.44	28.4 ± 5.758	25.41 ± 4.39	27.76 ± 5.82	28.65 ± 4.46
**Never smoker n (%)**	48 (54.5%)	22 (53.7%)	13 (56.5%)	6 (40.0%)	7 (77.8%)
**Current smoker n (%)**	2 (2.3%)	1 (2.4%)	1 (4.3%)	0 (0.0%)	0 (0.0%)
**Ex smoker n (%)**	38 (43.2%)	18 (43.9%)	9 (39.1%)	9 (60.0%)	2 (22.2%)
**P/Y (Current + ex)**	17.8 ± 14.1	15.79 ± 11.84	12.70 ± 16.12	22.00 ± 12.19	37.00 ± 21.21
**Early onset (year) n (%)**	20 (22.7%)	13 (31.7%)	5 (21.7%)	1 (6.7%)	1 (11.1%)
**Age of onset (years)**	33.39 ±16.56	36.67 ± 15.89	31.96 ± 19.50	32.71 ± 19.09	39.33 ± 24.41
**Comorbidities**					
**ASA intolerance n (%)**	16 (18.2%)	9 (22.0%)	5 (21.7%)	1 (6.7%)	1 (11.1%)
**Rhinitis n (%)**	68 (77.3%)	34 (82.9%) ^§§§^	21 (91.3%) ^§§§^	11 (73.3%) ^§^	2 (22.2%)
**Sinusitis (with or without polyps) n (%)**	50 (56.8%)	25 (61.0%)	17 (73.9%)	8 (53.3%)	6 (66.7%)
**Nasal polyposis n (%)**	33 (37.5%)	11 (26.8%)	10 (43.5%)	7 (46.7%)	5 (55.6%)
**Bronchiectasis n (%)**	8 (9.1%)	2 (4.9%)	2 (8.7%)	4 (26.7%) *^§^	0 (0.0%)
**GERD n (%)**	20 (22.7%)	5 (12.2%)	5 (21.7%)	6 (40.0%)	4 (44.4%) *
**OSAS n (%)**	4 (4.5%)	1 (2.4%)	1 (4.3%)	1 (6.7%)	1 (11.1%)
**Obesity n (%)**	24 (27.3%)	8 (19.5%)	11 (47.8%) *	3 (20.0%)	2 (22.2%)
**Diabetes n (%)**	7 (8%)	3 (7.3%)	1 (4.3%)	1 (6.7%)	2 (22.2%)
**Hypertension n (%)**	24 (27.3%)	11 (26.8%)	8 (34.8%)	4 (26.7%)	1 (11.1%)
**MI n (%)**	3 (3.4%)	1 (2.4%)	0 (0.0%)	0 (0.0%)	2 (22.2%)
**Heart failure n (%)**	4 (4.5%)	1 (2.4%)	0 (0.0%)	1 (6.7%)	2 (22.2%)
**Arrhythmias n (%)**	6 (6.8%)	4 (9.8%)	0 (0.0%)	1 (6.7%)	1 (11.1%)
**SAD n (%)**	10 (11.4%)	6 (14.6%)	3 (13.0%)	0 (0.0%)	1 (11.1%)
**VCD n (%)**	2 (2.3%)	0 (0.0%)	0 (0.0%)	0 (0.0%)	2 (22.2%)
**EGPA n (%)**	0 (0%)	0 (0.0%)	0 (0.0%)	0 (0.0%)	0 (0.0%)
**Osteoporosis n (%)**	12 (13.6%)	8 (19.5%)	2 (8.7%)	1 (6.7%)	1 (11.1%)
**Past pneumoniae n (%)**	15 (17%)	6 (14.6%)	5 (21.7%)	3 (20.0%)	1 (11.1%)
**ABPA n (%)**	2 (2.3%)	2 (4.9%)	0 (0.0%)	0 (0.0%)	0 (0.0%)
**Chronic pain n (%)**	4 (4.5%)	2 (4.9%)	1 (4.3%)	0 (0.0%)	1 (11.1%)
**Arthropathies n (%)**	6 (6.8%)	1 (2.4%)	3 (13.0%)	2 (13.3%)	0 (0.0%)
**Familiarity n (%)**	16 (18.2%)	7 (17.1%)	2 (8.7%)	4 (26.7%)	3 (33.3%)
**Atopy n (%)**	64 (72.7%)	41 (100.0%)	12 (52.2%) ****	7 (46.7%) ****	4 (44.4%) ****
**Monosesitize n (%)**	12 (13.6%)	5 (12.2%)	3 (13.0%)	2 (13.3%)	2 (22.2%)
**Polysensitized n (%)**	52 (59.1%)	35 (85.4%)	9 (39.1%) ***	7 (46.7%) **	1 (11.1%) ****
**Seasonal allergen n (%)**	52 (59.1%)	33 (80.5%)	10 (43.5%) **	8 (53.3%) *	1 (11.1%) ****/#
**Perennial allergen n (%)**	49 (55.7%)	35 (85.4%)	6 (26.1%) ****	4 (26.7%) ****	4 (44.4%) **
**Alternaria n (%)**	7 (7.95%)	6 (14.6%)	1 (4.3%)	0 (0.0%)	0 (0.0%)
**Aspergillus n (%)**	16 (18.18%)	11 (26.8%)	4 (17.4%)	0 (0.0%) *	1 (11.1%)
**Specific IgE n (%)**	13 (14.8%)	8 (19.5%)	4 (17.4%)	0 (0.0%)	1 (11.1%)
**Prick test n (%)**	6 (6.8%)	6 (14.6%)	0 (0.0%)	0 (0.0%)	0 (0.0%)
**Treatment/Clinical outcome**					
**BDP HFA dose, mcg**	702.30 ± 216.00	673.17 ± 244.97	650.00 ± 174.93	783.33 ± 154.35 *°	757.89 ± 216.84
**LABA n (%)**	88 (100%)	41 (100%)	23 (100%)	15 (100%)	9 (100%)
**LAMA n (%)**	33 (37.5%)	12 (29.3%)	10 (43.5%)	6 (40.0%)	5 (55.6%)
**Chronic OCS n (%)**	24 (27.3%)	9 (22%)	8 (34.8%)	4 (26.7%)	3(33.3%)
**OCS bursts ≥ 3/year n (%)**	44 (50%)	17 (41.5%)	15 (65.2%)	9 (60.0%)	3 (33.3%)
**OCS bursts ≥ 3/year and Chronic OCS n (%)**	15 (17.04%)	4 (9.7%)	6 (26.08%)	3 (20%)	2 (22.2%)
**OCS dependence n (%)**	53 (60.2%)	22 (53.7%)	17 (73.9%)	10 (66.7%)	4 (44.4%)
**Biologic switches n (%)**	16 (18%)	8 (19.5%)	5 (21.7%)	2 (13.3%)	1 (11.1%)
**ACT score**	17.65 ± 4.41	19.37 ± 2.97	16.69 ± 4.84 *	17.61 ± 4.98	15.42 ± 4.55 **
**Controlled (ACT ≥ 20) n (%)**	37 (42%)	26 (63.4%)	5 (21.7%) **	5 (33.3%)	1 (11.1%) **
**Not controlled (ACT ≤ 19) n (%)**	51 (58%)	15 (36.6%)	18 (78.3%) **	10 (66.7%)	8 (88.9%) **
**Activity limitations**	3.09 ± 1.24	3.19 ± 1.08	3.12 ± 1.24	3.44 ± 1.46	2.84 ± 1.21
**Nocturnal symptoms**	3.94 ± 1.40	4.52 ± 0.87	3.85 ± 1.46	3.82 ± 1.59	3.95 ± 1.58
**Exacerbations/year**	3.55 ± 2.94	3.15 ± 3.07	4.27 ± 2.91	4.00 ± 2.83	2.58 ± 1.95 °
**ER visits n (%)**	33 (37.5%)	13 (31.7%)	8 (34.8%)	7 (46.7%)	5 (55.6%)
**Intubation n (%)**	1 (1.1%)	1 (2.4%)	0 (0.0%)	0 (0.0%)	0 (0.0%)

BMI: Body mass index, P/Y: pack/years, ASA intolerance: Aspirin intolerance, GERD: gastroesophageal reflux disease, OSAS: obstructive sleep apnea syndrome, MI: myocardial infarction, SAD: social anxiety disorder; VCD: vocal cord dysfunction, EGPA: eosinophilic granulomatosis with polyangiitis, ABPA: allergic bronchopulmonary aspergillosis, BDP HFA dose: beclomethasone mcg equivalent dose hydrofluoroalkane, LABA: long acting beta agonist; LAMA: long-acting muscarinic antagonist, OCS: oral corticosteroids, ACT: asthma control test, and ER: emergency room. Significance vs. omalizumab: * < 0.05, ** < 0.001, *** < 0.0001, **** < 0.00001, significance vs. mepolizumab: ° < 0.05, significance vs. benralizumab: # < 0.05, and significance vs. dupilumab: § < 0.05, §§§ < 0.0001.

**Table 2:** Baseline functional and biological characteristics of patients treated with different biologics. Results are expressed as mean ±standard deviation.

Functional Parameters/Biomarkers					
	Overall	Omalizumab	Mepolizumab	Benralizumab	Dupilumab
	T0	T0	T0	T0	T0
**FVC abs. (L)**	2.75 ± 1.01	2.76 ± 1.04	2.79 ± 1.05	2.95 ± 0.99	2.37 ± 0.60 #
**FVC % pred.**	86.88 ± 17.98	89.02 ± 18.91	83.50 ± 16.67	93.56 ± 22.56	83.05 ± 19.70
** FEV_1_ abs. (L)**	1.701 ± 0.71	1.67 ± 0.63	1.72 ± 0.86	1.87 ± 0.75	1.32 ± 0.51 *#
** FEV_1_ % pred.**	65.50 ± 17.73	65.08 ± 15.71	62.88 ± 19.41	72.67 ± 21.08	57.21 ± 18.89 #
** IT abs.**	59.43 ± 12.34	59.19 ± 13.47	58.59 ± 11.70	60.24 ± 14.47	53.58 ± 13.11
** IT % pred.**	72.74 ± 14.78	69.50 ± 13.71	74.46 ± 15.65	75.29 ± 18.95	67.78 ± 15.90
** RV abs. (L)**	3.09 ± 1.11	3.08 ± 1.04	3.18 ± 1.03	3.22 ± 1.45	3.13 ± 1.14
** RV % pred.**	147.70 ± 47.37	159.11 ± 46.37	148.73 ± 45.27	139.72 ± 49.73	150.94 ± 44.80
** FVC post BD abs. (L)**	3.02 ± 1.16	3.03 ± 1.2	2.95 ± 1.24	3.22 ± 1.11	2.57 ± 0.66 #
**FVC Delta abs. post BD (L)**	0.30 ± 0.24	0.33 ± 0.28	0.27 ± 0.21	0.25 ± 0.22	0.23 ± 0.17
** FVC Delta % post BD**	11.11 ± 8.3	11.92 ± 9.24	9.12 ± 7.18	7.36 ± 7.23	8.05 ± 9.17
** FEV_1_ post abs. (L)**	1.93 ± 0.85	1.95 ± 0.76	1.82 ± 1.10	2.05 ± 0.85	1.53 ± 0.6 *#
**FEV_1_ Delta abs. post BD (L)**	0.23 ± 0.19	0.24 ± 0.17	0.23 ± 0.19	0.18 ± 0.22	0.21 ± 0.18
** FEV_1_ Delta % post BD**	15.01 ± 9.70	15.58 ± 8.35	15.66 ± 9.72	10.49 ± 12.76	16.16 ± 9.36
** DLCO %**	85.50 ± 20.16	87.71 ± 9.94	78.42 ± 23.13	85.38 ± 26.87	76.60 ± 13.94
**DLCO/Va %**	100.20 ± 22.13	102.86 ± 19.62	93.70 ± 20.64	101.30 ± 30.07	98.00 ± 22.40
**F_E_NO (ppb)**	40.34 ± 29.42	35.47 ± 27.81	52.73 ± 33.00	39.96 ± 24.39	31.13 ± 22.94 °
**Total IgE (UI/mL)**	215.50 ± 180.40	323.45 ± 261.19	307.65 ± 421.13	466.07 ± 461.11	327.75 ± 649.39
**Leucocytes absolute count (cells/mcl)**	8124.00 ± 2121.00	8190.00 ± 1951.47	7771.65 ± 1822.44	8248.75 ± 1923.05	9152.63 ± 2546.82
**Neutrophils (%)**	55.00 ± 10.00	55.12 ± 9.19	52.95 ± 10.77	54.54 ± 8.11	58.55 ± 11.33
**Neutrophils absolute count (cells/mcl)**	4513.00 ± 1676.00	4588.46 ± 1583.27	4058.47 ± 1566.19	4570.62 ± 1274.58	5266.84 ± 1930.38
**Eosinophils (%)**	5.90 ± 4.45	5.44 ± 4.06	7.02 ± 4.13	7.00 ± 5.57	3.59 ± 2.59 °°#*
**Eosinophils absolute count (cells/mcl)**	436.30 ± 294.70	426.79 ± 310.28	560.38 ± 312.03	563.89 ± 468.83	296.84 ± 193.16 °#*
**Fibrinogen (mg/dL)**	355.00 ± 94.42	356.62 ± 95.72	363.40 ± 112.49	327.29 ± 86.61	366.86 ± 79.42

FEV_1_: Forced expiratory volume in 1 s; FVC: forced vital capacity, Abs: absolute, Post BD: post bronchodilators, pred.: predicted, IT: Tiffenau index, RV: residual volume, DLCO: diffusion capacity for carbon monoxide, and FeNO: exhaled nitric oxide. Significance vs. omalizumab: * < 0.05, Significance vs. Mepolizumab: ° < 0.05, °° < 0.001, significance vs. benralizumab: # < 0.05.

**Table 3:** Distribution of expression of T2 biomarkers in patients treated with different biologics.

T2 Biomarkers					
(n)	Overall (88)	Omalizumab (41)	Mepolizumab (23)	Benralizumab (15)	Dupilumab (9)
**1–2 biomarker n (%)**	39 (44.3%)	20 (48.8%)	7 (30.4%)	5 (33.3%)	7 (77.7%) °#
**3–4 biomarkers n (%)**	49 (55.6%)	21 (51.2%)	16 (69.5%)	10 (66.6%)	2 (22.2%) °#

Significance vs. mepolizumab: ° < 0.05, significance vs. benralizumab: # < 0.05.

**Table 4:** “Intra-biologic” analysis of functional and biological characteristics of patients treated with a biologic therapy over years.

	Omalizumab					Mepolizumab					Benralizumab			Dupilumab	
	T0	T1	T2	T3	T4	T0	T1	T2	T3	T4	T0	T1	T2	T0	T1
**BDP HFA dose, mcg**	673.17 ± 244.97	662.50 ± 258.88	590.91 ± 271.99	558.06 ± 293.00	500.00 ± 258.20 *	650.00 ± 174.93	580.00 ± 261.41	600.00 ± 215.21	600.00 ± 181.50	688.89 ± 247.21	783.33 ± 154.35	766.67 ± 123.67	650.00 ± 232.99	757.89 ± 216.84	800.00 ± 240.37
**ACT score**	19.37 ± 2.97	20.70 ± 2.56 *	21.03 ± 3.48 *	20.83 ± 3.44	21.57 ± 2.92 *	16.69 ± 4.84	20.67 ± 3.41 °°	20.40 ± 4.67 °	21.83 ± 3.00 °°°°	22.22 ± 3.42 °°	17.61 ± 4.98	21.94 ± 4.28 #	21.00 ± 3.96	15.42 ± 4.55	20.00 ± 3.86 §§
**Activity limitation**	3.19 ± 1.08	4.05 ± 0.90 *	4.35 ± 1.00 **	4.17 ± 1.20 *	4.50 ± 1.16 *	3.12 ± 1.24	3.92 ± 0.97 °	3.95 ± 1.19 °	4.39 ± 0.78 °°°	4.56 ± 0.73 °°	3.44 ± 1.46	4.17 ± 1.25	4.38 ± 0.74 #	2.84 ± 1.21	3.95 ± 1.13 §
**Nocturnal symptoms**	4.52 ± 0.87	4.82 ± 0.50	4.76 ± 0.97	4.83 ± 0.38	4.93 ± 0.27	3.85 ± 1.46	4.83 ± 0.64 °	4.30 ± 1.38	4.67 ± 0.77 °	4.67 ± 1.00	3.82 ± 1.59	4.61 ± 1.04	4.88 ± 0.35 #	3.95 ± 1.58	4.26 ± 1.15
**Exacerbations/year**	3.15 ± 3.07	1.00 ± 1.47 ***	1.22 ± 1.70 **	1.68 ± 1.72 *	1.13 ± 1.58 **	4.27 ± 2.91	1.08 ± 1.35 °°°°	0.75 ± 0.91 °°°°	0.61 ± 0.70 °°°°	0.44 ± 0.73 °°°°	4.00 ± 2.83	0.89 ± 0.90 ##	1.12 ± 1.25 ##	2.58 ± 1.95	0.42 ± 0.96 §§§
**FVC abs. (L)**	2.76 ± 1.04	3.04 ± 1.12	2.94 ± 1.07	2.94 ± 1.07	2.78 ± 1.03	2.79 ± 1.05	3.02 ± 1.19	2.88 ± 0.86	3.39 ± 1.34	2.81 ± 0.72	2.95 ± 0.99	3.11 ± 1.11	3.44 ± 1.22	2.37 ± 0.60	2.54 ± 0.69
**FVC % pred.**	89.02 ± 18.91	97.72 ± 16.27 *	95.55 ± 16.57	97.63 ± 17.47 *	99.08 ± 18.92 *	83.50 ± 16.67	91.61 ± 25.16	99.12 ± 27.29 °	108.40 ± 16.15 °°°°	98.71 ± 18.51	93.56 ± 22.56	96.61 ± 23.09	105.00 ± 23.00	83.05 ± 19.70	85.71 ± 21.93
**FEV_1_ abs. (L)**	1.67 ± 0.63	1.87 ± 0.72	1.87 ± 0.63	1.90 ± 0.71	1.81 ± 0.73	1.72 ± 0.86	2.01 ± 1.04	1.82 ± 0.73	2.33 ± 1.15	1.89 ± 0.57	1.87 ± 0.75	2.11 ± 1.00	2.72 ± 0.95 #	1.32 ± 0.51	1.58 ± 0.65
**FEV_1_ % pred.**	65.08 ± 15.71	72.97 ± 17.64 *	75.30 ± 16.93 *	77.26 ± 19.49 *	76.70 ± 21.90 *	62.88 ± 19.41	73.62 ± 28.37	76.06 ± 28.78	86.73 ± 20.79 °°	81.43 ± 18.61 °	72.67 ± 21.08	79.06 ± 28.85	98.12 ± 20.57 #	57.21 ± 18.89	64.47 ± 19.23
**IT abs.**	59.19 ± 13.47	61.69 ± 9.19	63.43 ± 9.53	63.97 ± 11.01	61.21 ± 11.53	58.59 ± 11.70	61.18 ± 12.54	60.10 ± 12.97	64.33 ± 13.04	65.99 ± 8.27	60.24 ± 14.47	62.21 ± 14.24	72.00 ± 5.63 #	53.58 ± 13.11	59.56 ± 13.12
**IT % pred.**	69.50 ± 13.71	77.82 ± 11.10 *	78.85 ± 12.5 *	79.41 ± 14.3 *	75.96 ± 13.12	74.46 ± 15.65	76.77 ± 15.77	73.88 ± 16.09	80.67 ± 17.22	79.43 ± 8.89	75.29 ± 18.95	79.94 ± 19.59	94.14 ± 8.13 ##	67.78 ± 15.90	73.33 ± 15.43
**RV abs. (L)**	3.08 ± 1.04	2.63 ± 0.78	2.57 ± 1.06	2.79 ± 0.82	2.18 ± 0.85 *	3.18 ± 1.03	2.85 ± 0.97	3.08 ± 0.83	2.55 ± 0.56	2.44 ± 0.73	3.22 ± 1.4	2.78 ± 1.17	3.25 ± 2.08	3.13 ± 1.14	2.47 ± 0.07 §
**RV % pred.**	159.11 ± 46.37	127.15 ± 38.82 *	132.64 ± 44.90	139.33 ± 42.27	113.00 ± 28.21 **	148.73 ± 45.27	143.13 ± 51.42	154.36 ± 36.97	127.58 ± 31.77	121.20 ± 41.03	139.72 ± 49.73	142.71 ± 50.81	105.43 ± 46.24	150.94 ± 44.80	125.33 ± 41.85
**FVC post BD abs.(L)**	3.03 ± 1.21	3.48 ± 1.26	3.03 ± 0.63	2.99 ± 0.78	2.82 ± 1.23	2.95 ± 1.24	3.13 ± 1.08	3.02 ± 0.96	3.62 ± 1.29	3.21 ± 0.46	3.22 ± 1.11	3.30 ± 1.08	3.35 ± 1.52	2.57 ± 0.66	2.69 ± 0.73
**FVC Delta abs. post BD (L)**	0.33 ± 0.28	0.15 ± 0.21 *	0.22 ± 0.29	0.18 ± 0.17	0.16 ± 0.21	0.27 ± 0.21	0.15 ± 0.12	0.20 ± 0.22	0.17 ± 0.19	0.08 ± 0.03 °	0.25 ± 0.22	0.24 ± 0.33	0.16 ± 0.14	0.23 ± 0.17	0.19 ± 0.16
**FVC Delta % post BD**	11.92 ± 9.24	6.48 ± 9.08	8.95 ± 13.60	6.39 ± 7.20	4.00 ± 6.22 *	9.12 ± 7.18	4.93 ± 4.25	6.62 ± 5.84	5.75 ± 6.95	2.42 ± 1.25 °°	7.36 ± 7.23	5.87 ± 6.96	5.62 ± 3.74	8.05 ± 9.17	9.91 ± 8.58
**FEV_1_ post abs. (L)**	1.95 ± 0.76	2.06 ± 0.84	1.84 ± 0.49	1.99 ± 0.59	2.01 ± 0.79	1.82 ± 1.10	2.26 ± 0.95	2.01 ± 0.88	2.58 ± 1.10	2.38 ± 0.21	2.05 ± 0.85	2.20 ± 0.82	2.56 ± 1.24	1.53 ± 0.63	1.60 ± 0.62
**FEV_1_ Delta Post BD abs. (L)**	0.24 ± 0.17	0.17 ± 0.12	0.15 ± 0.15	0.16 ± 0.14	0.21 ± 0.17	0.23 ± 0.19	0.16 ± 0.10	0.10 ± 0.46	0.17 ± 0.16	0.13 ± 0.14	0.18 ± 0.22	0.17 ± 0.20	0.13 ± 0.15	0.21 ± 0.18	0.17 ± 0.06
**FEV_1_ Delta % post BD**	15.58 ± 8.35	10.60 ± 8.24	8.38 ± 7.67 *	9.39 ± 9.98	11.55 ± 6.71	15.66 ± 9.72	7.52 ± 2.91 °°	1.72 ± 33.20	8.37 ± 10.17	6.55 ± 7.31	10.49 ± 12.76	10.49 ± 9.79	6.13 ± 5.31	16.16 ± 9.36	13.75 ± 9.05
**DLCO %**	87.71 ± 9.94	98.75 ± 21.87	105.50 ± 17.68	92.60 ± 14.98	97.00 ± 22.00	78.42 ± 23.13	64.00 ± 46.67		88.20 ± 15.06		85.38 ± 26.87	92.67 ± 24.01	94.50 ± 17.68	76.60 ± 13.94	89.00 ± 19.37
**F_E_NO (ppb)**	35.47 ± 27.81	31.54 ± 21.89	42.64 ± 34.23	35.85 ± 17.31	48.11 ± 51.61	52.73 ± 33.00	56.87 ± 50.08	41.99 ± 30.12	59.75 ± 50.89		39.96 ± 24.39	50.84 ± 60.13	53.60 ± 40.48	31.13 ± 22.94	29.48 ± 17.17
**Total IgE (UI/mL)**	323.45 ± 261.19	792.33 ± 649.35 *	606.55 ± 419.06 *	557.97 ± 409.13 *	576.06 ± 519.86	307.65 ± 421.13	236.91 ± 152.03	197.13 ± 213.36	149.00 ± 49.50	641.95 ± 833.04	466.07 ± 461.11	613.67 ± 793.41		327.75 ± 649.39	221.05 ± 280.84
**Leukocytes absolute count (cells/mcl)**	8190.00 ± 1951.47	7357.42 ± 1952.10	7977.14 ± 3673.14	7351.15 ± 1679.39	7321.50 ± 2063.33	7771.65 ± 1822.44	7977.39 ± 1870.07	7325.56 ± 2491.86	7248.82 ± 2038.64	7220.00 ± 1464.66	8248.75 ± 1923.05	7551.76 ± 2973.69	6182.86 ± 1190.44 ##	9152.63 ± 2546.82	8011.25 ± 1772.18
**Neutrophils %**	55.12 ± 9.19	59.48 ± 11.54	58.88 ± 8.09	54.04 ± 7.84	56.07 ± 7.01	52.95 ± 10.77	57.99 ± 8.98	55.28 ± 10.03	60.73 ± 11.93	56.89 ± 7.43	54.54 ± 8.11	58.46 ± 10.16	49.07 ± 9.79	58.55 ± 11.33	53.83 ± 5.49
**Neutrophils absolute count (cells/mcl)**	4588.46 ± 1583.27	4246.47 ± 1556.59	5072.31 ± 2982.80	4062.31 ± 1195.96	4072.22 ± 1414.37	4058.47 ± 1566.19	4574.55 ± 1522.37	4115.00 ± 1994.25	4817.65 ± 2309.18	4085.71 ± 821.52	4570.62 ± 1274.58	4490.00 ± 2040.00	3115.00 ± 299.25 ##	5266.84 ± 1930.38	4304.50 ± 1610.92
**Eosinophils %**	5.44 ± 4.06	4.59 ± 3.71	5.17 ± 4.76	5.35 ± 3.44	5.03 ± 2.97	7.02 ± 4.13	1.43 ± 1.16 °°°°	1.50 ± 1.25 °°°°	1.29 ± 1.37 °°°°	1.04 ± 0.59 °°°°	7.00 ± 5.57	0.53 ± 2.00 ###	0.00 ± 0.00 ####	3.59 ± 2.59	5.94 ± 6.57
**Eosinophils absolute count (cells/mcl)**	426.79 ± 310.28	341.68 ± 271.28	363.29 ± 271.66	396.40 ± 231.73	403.12 ± 218.90	560.38 ± 312.03	108.26 ± 79.98 °°°°	105.00 ± 81.47 °°°°	85.88 ± 81.17 °°°°	74.29 ± 38.23 °°°°	563.89 ± 468.83	47.06 ± 188.94 ###	0.00 ± 0.00 ####	296.84 ± 193.16	527.06 ± 613.29
**Fibrinogen (mg/dl)**	356.62 ± 95.72	344.33 ± 100.47	330.67 ± 73.95	429.40 ± 88.71		363.40 ± 112.49		369.25 ± 95.12	404.22 ± 101.63	419.75 ± 129.64	327.29 ± 86.61	327.67 ± 92.39	294.00 ± 75.36	366.86 ± 79.42	382.50 ± 98.99

BDP HFA dose: beclomethasone mcg equivalent dose hydrofluoroalkane, FEV_1_: forced expiratory volume in 1 s; FVC: forced vital capacity, Abs: absolute, Post BD: post bronchodilators, pred.: predicted, IT: Tiffenau index, RV: residual volume, DLCO: diffusion capacity for carbon monoxide, and FeNO: exhaled nitric oxide. Results are expressed as mean ±standard deviation significance vs. T0 in omalizumab: * < 0.05, ** < 0.001,*** < 0.0001, significance vs. T0 in mepolizumab: ° < 0.05, °° < 0.001, °°° < 0.0001, °°°° < 0.00001, significance vs. T0 in benralizumab: # < 0.05, ## < 0.001, ### < 0.0001, #### < 0.00001, and significance vs. T0 in dupilumab: § < 0.05, §§ < 0.001, §§§ < 0.0001,. Empty cells: not enough values for calculation.

**Table 5:** “Inter-biologic” analysis of functional and biological characteristics of patients treated with a biologic therapy over years.

	Omalizumab				Mepolizumab				Benralizumab		Dupilumab
	Delta T1	Delta T2	Delta T3	Delta T4	Delta T1	Delta T2	Delta T3	Delta T4	Delta T1	Delta T2	Delta T1
**BDP HFA dose, mcg**	−17.50 ±169.29	−69.70 ±251.85	−87.10 ±301.93	−100.00 ±263.00	−72.00 ±279.17	−40.00 ±270.28	−16.67 ±214.89	55.56 ±278.89	−16.67 ±161.79	−37.50 ±346.15	42.11 ± 254.55
**ACT score**	1.40 ± 3.42	1.48 ± 3.78	1.17 ± 3.75	1.96 ± 3.97	3.88 ± 3.78	3.45 ± 4.66	4.22 ± 4.98 *	6.33 ± 7.07	4.33 ± 5.76 *	5.12 ± 3.09 *	4.58 ± 4.03 **
**Activity limitation**	0.81 ± 1.36	1.14 ± 1.56	0.77 ± 1.24	1.38 ± 1.19	0.75 ± 1.07	0.75 ± 1.33	1.06 ± 1.30	1.67 ± 1.41	0.72 ± 1.71	1.12 ± 1.25	1.11 ± 1.05
**Nocturnal symptoms**	0.29 ± 1.06	0.07 ± 1.07	0.08 ± 0.76	0.50 ± 0.76	0.92 ± 1.35	0.30 ± 1.38	0.67 ± 1.46	0.78 ± 1.86	0.76 ± 1.64	1.71 ± 1.38 *°	0.32 ± 1.38
**Exacerbations/year**	−2.28 ±2.80	−2.06 ± 3.74	−1.90 ±3.32	−2.57 ± 3.54	−3.38 ±2.63	−3.55 ±2.31	−4.00 ±3.22 *	−4.78 ±3.46	−3.11 ±2.78	−4.00 ±2.78	−2.16 ±1.61 *
**FVC abs. (L)**	0.24 ±0.39	0.22 ±0.38	0.26 ±0.46	0.16 ±0.64	0.15 ±0.54	0.14 ±0.47	0.29 ±0.44	−0.02 ± 0.31	0.16 ±0.47	0.26 ±0.49	0.15 ±0.39
**FVC % pred.**	6.94 ±15.91	5.55 ±14.80	8.76 ±16.62	8.04 ±23.81	6.57 ±21.27	14.94 ±23.08	18.53 ±16.82	17.29 ±22.34	3.06 ±14.95	8.14 ±14.58	3.47 ±15.99
**FEV_1_ abs. (L)**	0.19 ±0.42	0.23 ±0.35	0.28 ±0.39	0.19 ±0.47	0.21 ±0.45	0.14 ±0.40	0.31 ±0.45	0.04 ±0.29	0.24 ±0.46	0.41 ±0.47	0.23 ±0.46
**FEV_1_ % pred.**	7.17 ±18.52	9.93 ±13.72	13.60 ±16.68	12.23 ±20.58	9.50 ±17.37	12.94 ±19.21	16.00 ±16.27	13.86 ±18.40	6.39 ±14.97	13.12 ±15.53	7.35 ±19.54
**IT abs. (L)**	2.28 ±10.81	4.67 ±9.42	5.75 ±10.92	4.18 ±10.99	2.38 ±4.56	1.44 ±6.38	2.26 ±4.69	2.53 ±5.71	2.28 ±5.47	3.00 ±7.14	5.27 ±10.61
**IT % pred.**	6.67 ±13.42	9.46 ±11.87	12.75 ±11.80	7.88 ±12.41	2.82 ±6.62	−0.06 ±8.76 *	1.73 ± 7.82 **	−2.14 ±7.67 *	3.50 ±7.31	4.43 ±8.92	5.00 ±8.43
**RV abs. (L)**	−0.37 ±1.33	−0.29 ±0.68	−0.86 ±0.88	−0.94 ±0.47	−0.77 ±1.55	0.34 ±0.76	−0.37 ±0.47		−0.12 ±0.62	−0.29 ±0.24	−0.52 ±1.00
**RV % pred.**	−35.08 ±59.49	−9.50 ±36.03	−42.29 ±38.81	−54.00 ±17.11	−12.00 ±55.61	8.60 ±32.42	−17.80 ±19.69		10.57 ±46.88 *	−17.29 ±16.10	−21.40 ±35.64
**FVC post BD abs. (L)**	0.16 ±0.25	0.22 ±0.41	0.24 ±0.60	0.32 ±0.86	0.28 ±0.55	0.14 ±0.47	−0.03 ±0.53		0.11 ±0.33	0.19 ±0.37	−0.04 ±0.47
**FVC Delta abs. post BD (L)**	−0.08 ±0.19	−0.12 ±0.23	−0.25 ±0.30	−0.36 ±0.52	−0.04 ±0.24	0.01 ±0.17	−0.09 ±0.07		−0.01 ±0.49	−0.29 ±0.26	−0.02 ±0.27
**FVC Delta % post BD**	−3.25 ±6.19	−0.07 ±18.79	−9.68 ±7.88	−6.86 ±9.04	−3.19 ±8.45	1.18 ±8.32	−2.62 ±2.29		−2.42 ±11.01	−9.49 ±9.19	5.33 ±8.18 *
**FEV_1_ post abs. (L)**	0.07 ±0.35	0.12 ±0.16	0.41 ±0.53	0.52 ±0.66	0.27 ±0.51	0.11 ±0.48	0.01 ±0.41		0.14 ±0.30	0.28 ±0.27	0.13 ±0.48
**FEV_1_ Delta post BD abs. (L)**	−0.02 ±0.17	−0.03 ±0.09	−0.04 ±0.14	−0.02 ±0.08	−0.07 ±0.15	−0.15 ±0.42	−0.10 ±0.12		−0.02 ±0.29	−0.18 ±0.18	−0.00 ±0.16
**FEV_1_ Delta % post BD**	−1.33 ±10.02		17.00 ±5.66		2.00 ±18.38		−0.67 ±3.06		−12.50 ±17.68		−8.00 ±11.31
**DLCO %**	−1.42 ±8.01	−4.22 ±8.82	−7.19 ±10.01	−5.29 ±5.61	−6.75 ±9.06	−17.80 ±36.10	−4.87 ±6.36		0.63 ±15.24	−8.10 ±8.80	−0.23 ±10.56
**F_E_NO (ppb)**	−1.34 ±23.16	4.77 ± 36.86	−9.32 ±27.86	4.11 ±46.58	4.36 ±45.94	−7.94 ±21.08	4.61 ±31.11		13.68 ±58.70	−12.65 ±44.47	−0.61 ±29.36
**Total IgE (UI/mL)**	433.87 ± 469.39	244.41 ± 296.61	280.61 ± 267.28	336.62 ± 362.71	−150.81 ±335.34 *	−510.51 ±727.59	37.50 ±26.16 *	−245.20 ±361.76	138.75 ± 425.38		
**Leukocytes absolute count (cells/mcl)**	−613.20 ±1861.53	637.65 ±3656.95	−202.00 ±1700.98	−103.33 ±2191.77	235.52 ±2347.21	−25.72 ±3029.60	−591.18 ±2212.59	−637.14 ±1793.19	−359.33 ±2304.89	−531.43 ±933.82	−1332.50 ±2656.25
**Neutrophils %**	2.65 ± 6.80	5.56 ± 4.21	1.35 ± 4.89	−2.47 ±1.56	2.09 ±12.31	−2.95 ±11.22 *	4.55 ±9.29	−6.78 ±8.06	5.71 ±10.29	−3.03 ±11.69	−4.32 ±9.67 *#
**Neutrophils absolute count (cells/mcl)**	14.62 ± 1092.97	512.86 ± 557.37	8.00 ± 505.64		376.92 ± 2331.59	−546.70 ±2450.23	507.78 ± 2400.87	−1500.00 ± 608.11	218.00 ± 1553.83	−631.67 ±1050.17 *	−1056.75 ±2574.46
**Eosinophils %**	−1.33 ±3.15	−1.87 ±3.53	−2.66 ±4.00	−2.03 ±3.08	−5.70 ±4.39 **	−5.83 ±4.07 *	−6.93 ±3.98 **	−6.27 ±5.19	−6.49 ±6.79 *	−5.94 ±2.89 *	2.49 ±6.97 °°##*
**Eosinophils absolute count (cells/mcl)**	−94.72 ±236.97	−128.50 ±349.03	−128.50 ±261.76	−125.38 ±298.07	−463.91 ±329.31 ****	−471.50 ±308.17 **	−537.65 ±312.28 **	−465.71 ±338.62 *	−520.59 ±560.21 **	−367.14 ±148.74 *	231.18 ±674.46 °°°##
**Fibrinogen (mg/dL)**	0.00 ±0.00	16.75 ±25.58	54.00 ±119.25			22.88 ± 46.77	−20.00 ±23.61	−38.00 ±11.31	1.64 ± 30.03	15.00 ± 41.01	−38.33 ±44.66

BDP HFA dose: beclomethasone mcg equivalent dose hydrofluoroalkane, FEV_1_: forced expiratory volume in 1 s; FVC: forced vital capacity, Abs: absolute, post BD: post bronchodilators, pred.: predicted, IT: Tiffenau index, RV: residual volume, DLCO: diffusion capacity for carbon monoxide, and FeNO: exhaled nitric oxide. Values represent the variation in parameters from each time point (e. T1, T2, T3, and T4) to T0 (paired Delta). Delta are expressed as mean ± standard deviation significance vs. omalizumab: * < 0.05, ** < 0.001, **** < 0.00001, significance vs. mepolizumab: ° < 0.05, °° < 0.001, °°° < 0.0001, significance vs. benralizumab: # < 0.05, ## < 0.001. Empty cells: not enough values for calculation.

## Data Availability

Data are available on request from the authors.

## Supplementary Materials

The following supporting information can be downloaded at https://www.mdpi.com/article/10.3390/jcm13164750/s1, Table S1: Regional criteria for prescription of biologic treatment; Table S2: Number of evaluated patients at T1, T2, T3, T4 for each biologics and the total number of patients at each Time point; Table S3: Number of evaluated patients at T1, T2, T3, T4 for each biologics and the total number of patients at each Time point. Figure S1: Number and sequence of treatments for all biologic switchers (N = 16).
