# Supplementary material for: Biologics in T2 Severe Asthma: Unveiling Different Effectiveness by Real-World Indirect Comparison
Source: J Clin Med. 2024 Aug 13;13(16):4750. doi: 10.3390/jcm13164750 (PMC11355661; doi:10.3390/jcm13164750)

Table S1: Regional criteria for prescription of biologic treatment

REGIONAL PRESCRIPTIVE CRITERIA	
Omalizumab	≥ 6 years of age<br><br>***All the following criteria must be present:***<br><br>• Severe persistent allergic asthma for more than 12 months not controlled by maximally tolerated doses of inhaled glucocorticoids and long-acting beta two agonists<br>• Evidence of positivity for a perennial disease allergen (Prick test or specific IgE)<br>• Total serum IgE [IgE ≥ 76 IU/ml (adolescents and adults) or IgE ≥ 200 IU/ml (children) at treatment initiation] and specific IgE or positive skin test for a perennial allergen<br>• Respiratory function: (FEV_1 < 80%) at the start of treatment<br>• Poor asthma control<br>• Anti-asthmatic therapy at the time of prescription
Mepolizumab	≥ 6 years of age<br><br>• >150 currant eosinophils/mmc value in the absence of systemic steroid treatment and at least one value >300 eosinophils/mmc in the previous year<br>AND<br><br>• at least 2 exacerbations of asthma despite maximal inhaled therapy (treated with systemic steroids or requiring hospitalization) in the previous 12 months (Step 4-5 of GINA, from age 12) or despite maximally tolerated inhaled therapy (age 6 to 11)<br><br>OR<br><br>• Received continuous oral steroid therapy in addition to maximal inhaled therapy for at least 6 months in the past year (condition only applies to adults 18 years of age and older)
Benralizumab	≥ 18 years of age<br><br>• >300 eosinophils/mmc value in the absence of systemic steroid treatment<br><br>AND<br><br>• at least 2 exacerbations of asthma despite maximal inhaled therapy (treated with systemic steroids or requiring hospitalization) in the previous 12 months<br><br>OR<br><br>• Received continuous oral steroid therapy in addition to maximal inhaled therapy in the past year
Dupilumab	≥ 12 years of age<br><br>• >150 eosinophils/mmc value<br><br>OR<br><br>• > 25 ppb FeNO value<br><br>AND

	• at least 2 exacerbations of asthma despite maximal inhaled therapy, treated with systemic steroids or requiring hospitalization, in the previous 12 months (Step 4-5 of GINA)<br><br>OR<br><br>• Received continuous oral steroid therapy in addition to maximal inhaled therapy for at least 6 months in the past year

Table S2: Number of evaluated patients at T1, T2, T3, T4 for each biologics and the total number of patients at each Time point.

Biologic	N patients Baseline (T0)	T1	T2	T3	T4
**Omalizumab**	41	40	33	30	25
**Mepolizumab**	26	26	20	19	9
**Benralizumab**	18	18	8	1	-
**Dupilumab**	19	18	-	-	-
**N total biologic treated patients at each time point**	104	102	61	50	34

Table S3: Number of evaluated patients at T1, T2, T3, T4 for each biologics and the total number of patients at each Time point.

Biologic	OCS	1st year	2nd year	3rd year	4th year
**Omalizumab**	**withdrawal**	6 (66.6%)	6 (85.71%)	4 (66.6%)	3 (60%)
	**continued**	3 (33.3%)	1 (14.28%)	2 (33.3%)	2 (40%)
**n patients (%)**		9 (29.03%)	7 (38%)	6 (46.1%)	5 (62.5%)
**Mepolizumab**	**withdrawal**	7 (63.63%)	5 (71.42%)	6 (100%)	3 (100%)
	**continued**	3 (27.27%)	1 (14.28%)	-	-
	**50% reduction**	1 (9.09%)	1 (14.28%)	-	-
**n patients (%)**		11 (35.48%)	7 (38%)	6 (46.1%)	3(37.5%)
**Benralizumab**	**withdrawal**	1 (25%)	4 (100%)	1 (100%)	-

	**continued**	1 (25%)	-	-	-
	**50% reduction**	2 (50%)	-	-	-
**n patients (%)**		4 (12.90%)	4 (22%)	1 (100%)	-
**Dupilumab**	**withdrawal**	2 (28.57%)	-	-	-
	**continued**	3 (42.85%)	-	-	-
	**50% reduction**	2 (28.57%)	-	-	-
**n patients (%)**		7 (22.58%)			

Figure S1: Number and sequence of treatments for all biologic switchers (N=16)

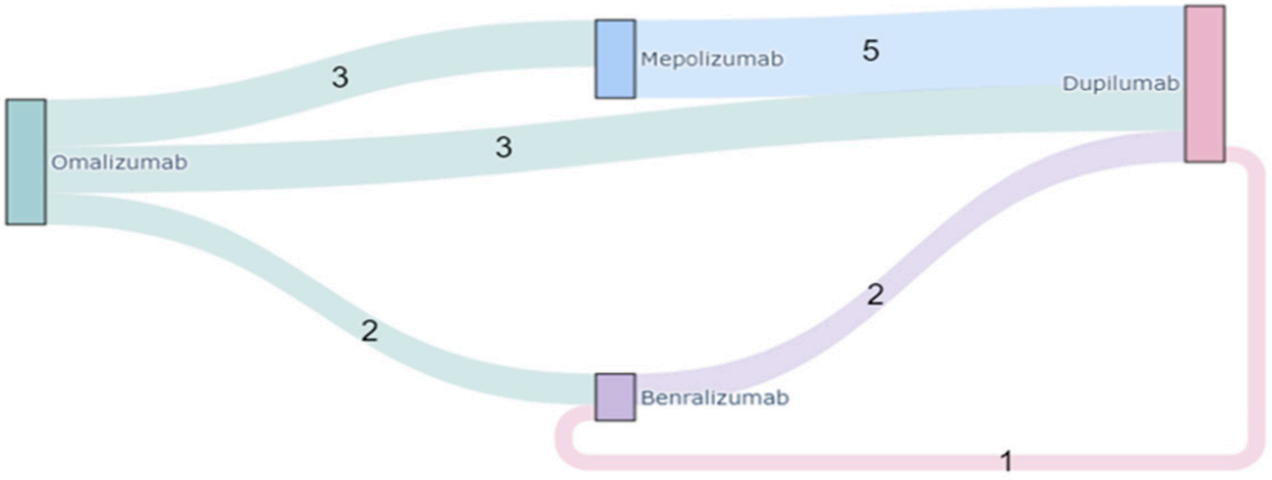

Supplement: Supplementary file 1 [file jcm-13-04750-s001.zip › jcm-3119634-supplementary.pdf]
